# The influence of wisdom tooth impaction and occlusal support on mandibular angle and condyle fractures

**DOI:** 10.1038/s41598-021-87820-9

**Published:** 2021-04-16

**Authors:** Hesham Mohammed Al-Sharani, Zhang Bin, Mubarak Ahmed Mashrah, Endi Lanza Galvão, Essam Ahmed Al-Moraissi, Maged Ali Al-Aroomi, Karim Ahmed Sakran, Saulo Gabriel Moreira Falci

**Affiliations:** 1 Department of Maxillofacial Surgery, Faculty of Dentistry, Ibb University, Ibb, Yemen; 2 Post-Graduate Program in Maxillofacial Surgery. First Affiliated Hospital, School of Stomatology, Jinzhou Medical University, Jinzhou, China; 3 Key Laboratory of Oral Medicine, Guangzhou Institute of Oral Disease, Stomatology Hospital of Guangzhou Medical University, Guangzhou, Guangdong, PR China; 4 Post-Graduate Program in Dentistry, Department of Dentistry, Oral and Maxillofacial Section, Faculty of Basic Sciences and Health, Federal University of Vales Jequitinhonha and Mucuri, Diamantina, Minas Gerais Brazil; 5 Faculty of Dentistry, Thamar University, Thamar, Yemen; 6 Department of Oromaxillofacial-Head and Neck Surgery, School and Hospital of Stomatology, China Medical University, Shenyang, China; 7 State Key Laboratory of Oral Diseases, National Clinical Research Center for Oral Diseases, and Department of Oral and Maxillofacial Surgery, West China Hospital of Stomatology, Sichuan University, Chengdu, China; 8 Department of Maxillofacial Surgery, School of Stomatology, Harbin Medical University, Harbin, China

**Keywords:** Oral anatomy, Oral diseases, Trauma

## Abstract

This study aimed to analyze the relationship of the occlusal support together with the lower third molars to the mandibular fractures of the angle and condyle among patients in our medical institutions. This was a retrospective study that reviewed the medical records and radiographs of all patients treated for mandibular fractures from 2015 to 2019. The data collected by using picture archiving and communicating system. Only records with mandibular angle or condyle fractures were included. The dependent variable was the presence of the fractures of the mandibular angle or condyle. The independent variables were epidemiological data, third molar characteristics, existence or absence of occlusal support. The data was analyzed through Univariate logistic regression and multivariate logistic regression. From a total of 187 mandibular fractures, 44 presented mandibular angle fracture and 29 shown condyle fractures. The average age was 40.34 ± 13.47 years. The absence of occlusal support increased the chance of condyle fractures by 5.1 times (95% CI 1.61–17.29). The lack of occlusal support is more associated with condyle fractures than the presence of occlusal support, regardless of third molar presence and characteristics and other variables evaluated.

## Introduction

The lower jaw is the hardest and largest facial bone; yet, it is more susceptible to fracture than other facial bones^1^. Fractures in this area belong to the lower jaw's exposed and protruded posture, along with its partial movement and embracement of the lower teeth, which may add local weak areas^2^. The diverse densities of the mandible with the bow U-shape indicates another reason to increase its fragility. Therefore, mandibular fractures MF take place as twice as maxillofacial fractures about 35–65%^1^. Earlier studies revealed that the lack of bone support and the presence of anatomical areas of weakness such as the existence of the lower teeth, foramina and neck of the condyle might explain the increased prevalence of mandibular fractures^3^. Pathological changes such as tumors, pre-apical lesions, and teeth impactions also describe an extra cause of fragility to the lower jaw. The fracture site in the lower jaw typically depends on various factors, including the specific point of force, the anatomical structure, and the direction and severity of the traumatic impact^4^.

The condyle and angle are the mandibular regions most likely to be fractured, presenting a prevalence ranging from 30 to 52% and 25 to 33%, respectively^1^. The leading cause of the condyle fractures is the fragility in the neck region due to its thin bony thickness. In comparison, the mandibular angle is quite thick, but furtherly weakened by the emergence of bone segments with different densities as the angle is estimated to be the line where the ramus and body meet^5,6^. Several investigations have intensely focused on the mandibular third molar M3s as a research spot in relation to the fractures of the mandibular angle and condyle. The majority of these studies showed that the existence of lower M3 might influence the mandibular angular fractures while its absence may influence fractures toward the mandibular condyle^7–10^. However, their findings were discordant^11^. Other studies noticed an association between the occlusal support and the occurrence of both fractures of the lower jaw angle and condyle^12,13^. Recent studies in the same field have gone further to examine the relation of these fractures (angle and condyle) not only with the lower third molar existence or absence but to its different positions and angulations^13,14^.

Four years ago, two comprehensive meta-analyses about third molar and mandibular angle and condyle fractures was published^7,8^. This systematic review included 35 and 13 papers, respectively. However, in the studies the occlusal support was not addressed. Thus, even that there are many papers about this issue, how the occlusal support influence in the results of mandibular angle and condyle fractures is still unknown. Up to date, there are few studies that discussed the prevalence of angular and condylar fractures in association with the occlusal support that has been previously available. In this study, it is carefully planned to examine these fractures independently with an eye opened to diagnose their relations to the occlusal support in one hand and the lower third molar’s status of impaction in the other.

## Methods

This is a cross-sectional study that was carried out to know the relationship between occlusal support, with the presence of M3s in its different characteristics to the fractures of the mandibular angle and condyle. This study was approved by the Jinzhou Medical University Ethics Committee (A2018-0101H) and all experiments were performed in accordance with relevant guidelines and regulations. Data was evaluated through the review of the clinical records and radiographs of patients in the department of oral and maxillofacial surgery at the three affiliated hospitals, during the period 2015–2019. The informed consent was obtained from all participants.

The study population involved all patients with mandibular fractures managed throughout the study period. The records which manifested mandibular condyle or angle fractures were selected for our analysis. Those patients who subjected to the criteria of inclusion were added. The data was collected using PACS (picture archiving and communicating system). Inclusion criteria were the records of patients with well-diagnosed mandibular angle or condyle fractures, with age over 17 years holding permanent dentition, and patients presented without comminuted mandibular fractures and pan-facial fractures, and records of dentulous or partially edentulous patients.

**Figure 1:**
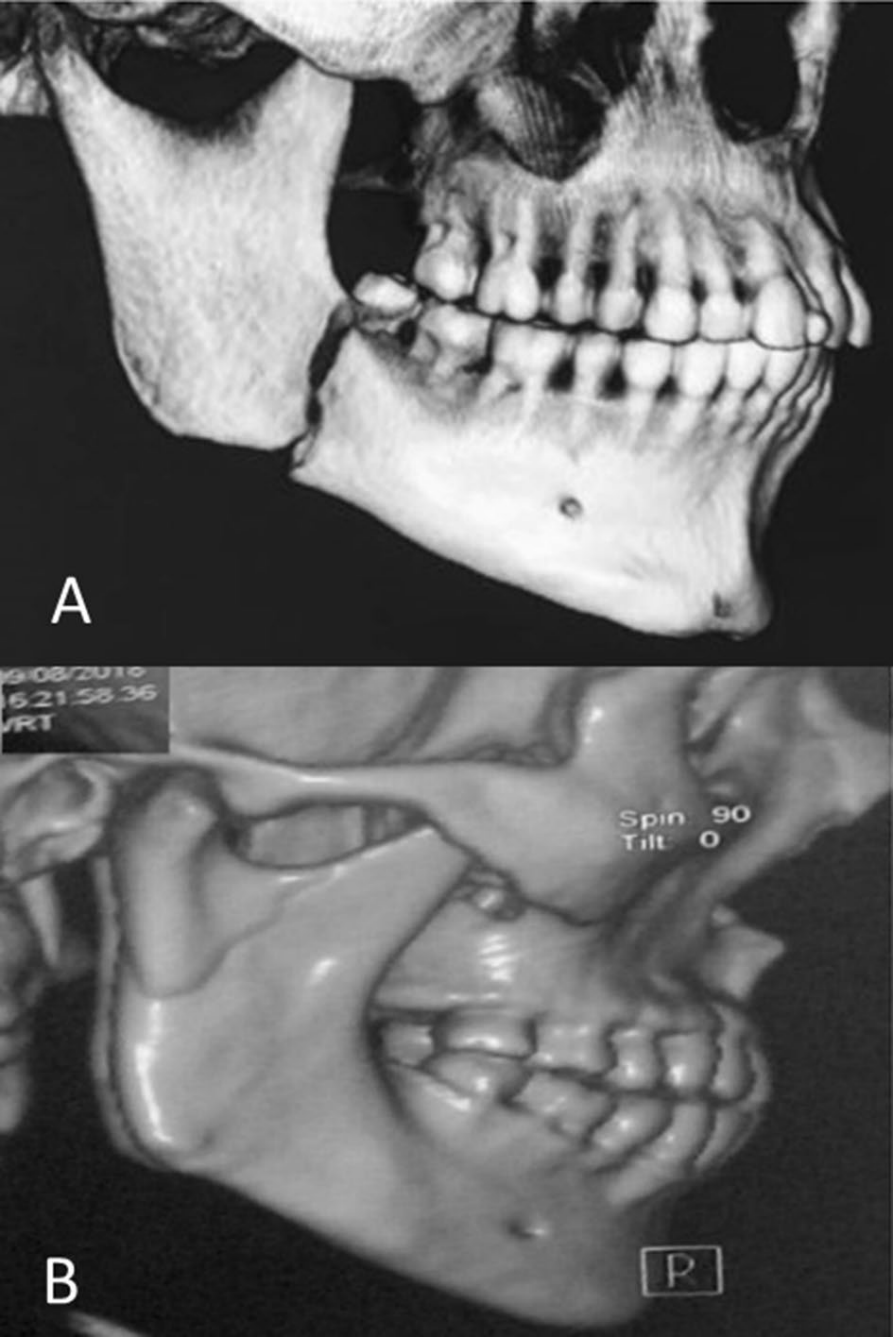
(**A**) Mandibular angle fracture; (**B**); Condylar fracture.

From the records included, the following data were collected:

1. Epidemiological data (gender, age and etiology)
2. Characteristics of mandibular angle/condyle fracture (isolated or associated with other sites as the body, symphysis, parasymphysis, ramus, and alveoli) (Fig. 1)
3. Third molar characteristics (presence or absence, M3 root configuration, vertical depth according to Pell & Gregory´s classification^15^, and M3 angulations based on Winter´s classification^16^
4. Occlusal support characteristics (presence or absence, partially edentulous, undiagnosed cases).

The dependent variable was the presence of mandibular angle or condyle fractures. The independent variables were epidemiological data, third molar characteristics, existence or absence of occlusal support.

Angular fractures are defined as the fractures situated backward to the lower second molar until the junction of the body and rami in the retromolar area. The condylar fractures are the fractures superior to the line determined from the condylar notch to the posterior margin of the ascending ramus^16^.

The occlusal support is defined based on the existence, or lack of normal occlusion in the region of the three molars^12^ M3 position types is according to Pell and Gregory’s Classification that assesses the relation of the lower third molar to the lower second molar and to evaluates the vertical depth of M3 within the ramus M3 angulation was assessed based on Winter’s Classification that assesses the angulation of M3 in relation to the apico-occlusal level of the lower M2.

Descriptive analysis was used to describe the sociodemographic and clinical features. Percentages and frequencies were applied for the categorical independent variables. The dependent variable was the type of mandibular fractures (angle or condyle), while the independent variables were screened by univariate analysis, and those with a *P* value less than 20% in this test were included in the multivariate regression analysis. The stepwise method assisted in selecting the variables to be included in the final model using Akaike Information Criterion (AIC). The fit of the model was assessed based on the *Hosmer–Lemeshow* test. Thus, the Odds Ratios (ORs) and 95% confidence intervals (CIs) for the types of mandibular fractures were calculated. Collinearity statistics were used to assess the possible collinearity between covariates. R software also used in data analysis (readxl and car packages), and *P* < 0.05 was regarded as significant^7,8^.

### Ethical approval

This study was approved by the ethical committee of the University of the first and second authors (A2018-0101H).

## Results

A total of 187 records of mandibular fractures were analyzed. Thirty-nine percent (73) presented a mandibular angle or condyle fracture. From this, 60.3% (44) were mandibular angular fractures. Males represented 78.1% of the study population. Moreover, 75.9% of condylar fractures were also seen in men. The average age was 40.34 ± 13.47 ranging from (17 to 65) years. The peak prevalence of both angular and condylar fractures in both genders was between the age of 35–50 years (38.4%). The description of the sociodemographic and clinical characteristics according to the type of mandibular fracture through a univariate analysis was present in Table 1.

**Table 1:** Univariate Analysis of Related Factors of the mandibular fractures (angle and condyle fractures).

Variables	Angle fracture (n = 44)	Condyle fracture (n = 29)	*P* value	OR (CI 95%)
**Gender**				
Male	35 (79.5%)	22 (75.9%)	0.710	1
Female	9 (20.5%)	7 (24.1%)		1.23 (0.39 – 3.80)
**Age groups**				
17–34 Age groups	12 (27.3%)	11 (37.9%)	0.547	1
35–50 Age groups	17 (38.6%)	11 (37.9%)		0.75 (0.22 – 2.15)
51–65 Age groups	15 (34.1%)	7 (24.1%)		0.50 (0.14 – 1.69)
**Etiology**				
Road traffic accidents	22 (50.0%)	10 (34.5%)	0.021*	1
Falls	13 (29.5%)	18 (62.1%)		3.04 (1.10 – 8.83)*
Assaults	7 (15.9%)	1 (3.4%)		0.31 (0.01 – 2.12)
**Status of fractures**				
Isolated fractures	27 (61.4%)	12 (41.4%)	0.094	1
Associated fractures	17 (38.6%)	17 (58.6%)		2.25 (0.87 – 5.97)
**Associated site**				
Body	5 (29.4%)	3 (17.6%)	0.728	–
Symphysis	5 (29.4%)	5 (29.3%)		**–**
Parasymphysis	3 (17.6%)	4 (23.5%)		**–**
Angle	1 (5.9%)	1 (5.9%)		**–**
Condyle	1 (5.9%)	4 (23.5%)		**–**
Ramus	1 (5.9%)	0		**–**
Alveoli	1 (5.9%)	0		**–**
**M3**				
Present	31 (70.5%)	13 (44.8%)	0.026*	1
Absent	13 (29.5%)	16 (55.2%)		2.93 (1.11 – 7.98)*
**Root configuration**				
Unclear cases	7 (22.6%)	2 (15.4%)	0.570	1
Separate roots	10 (32.3%)	4 (30.8%)		1.40 (0.20–12.19)
Fused roots	8(25.8%)	2 (15.4%)		0.87 (0.08–8.94)
Single root	6 (19.4%)	5 (38.5%)		2.91 (0.44–26.12)
**Vertical depth of M3**				
Type A	10 (32.3%)	4 (30.8%)	0.367	1
Type B	15 (48.4%)	4 (30.8%)		0.66 (0.12–3.41)
Type C	6 (19.4%)	5 (38.5%)		2.08 (0.39–11.69)
**M3 horizontal impaction**				
Class I	17 (54.8%)	2 (15.4%)	0.035*	1
Class II	10 (32.3%)	6 (46.2%)		5.10 (0.96–39.65)
Class III	4 (12.9%)	5 (38.5%)		10.62 (1.66–97.33)*
**M3 angulations**				
Mesioangular	11 (35.5%)	4 (30.8%)	0.793	1
Distoangular	5 (16.1%)	1 (7.7%)		0.55 (0.02–5.07)
Vertical	8 (25.8%)	5 (38.5%)		1.71 (0.34–9.03)
Horizontal	7 (22.3%)	3 (23.1%)		1.17 (0.18–7.04)
**Occlusal support**				
Occlusal support present	29 (76.3%)	8 (30.8%)	< 0.001*	1
Occlusal support absent	9 (23.7%)	18 (69.2%)		7.25 (2.46–23.45)*

*Means significant statistical difference.

In univariate analysis, there was an association between type of mandibular fracture (angle or condyle) and etiology, M3 presence or absence, M3 horizontal impaction, and the presence or absence of occlusal support.

Class I type B of M3 impactions are correlated with angle fractures while Class II type C impactions are associated with condyle fractures.

In a multivariate logistic regression analysis, after including the variables that contribute significantly to the model. The absence of occlusal support increases the chances of condyle fracture by 5.1 times (95%CI 1.61 – 17.29) (Table 2).

**Table 2:** Multiple Logistic Analysis of Odds Ratio of mandibular fractures (angle and condyle fractures).

Variables	b	SE(b)	*P*	OR	95% CI
Occlusal support absent	1.6305	0.5999	0.006570*	5.10	1.61–17.29
Falls	0.9637	0.6022	0.109526	2.62	0.79–8.67

*Means significant statistical difference.

## Discussion

The present study has purposed to step further with determining whether the existence or absence of occlusal support and lower M3 (position, angulation, and roots configurations) has a role in influencing the mandibular angular/condylar fractures.

This study asserted that men are experiencing angular and condylar fractures in higher proportions than women with (4:1) Male to Female Ratio which is in a convergent path with other documented studies on mandibular fractures, in China (4.1:1) and Brazil (5.4:1). The given male to female ratio is a precise index to the risky activities that men usually involved in comparison to women^5,9,17^.

Mandibular fractures generally induced by a variety of factors including biomechanical factors such as bone density, the applied force and the course of its impact as well as occlusal loading patterns; and pathological factors including preapical lesions, bone pathosis, teeth in the fracture line and other systemic illnesses^12,18^. The usual causes for mandibular fractures in several past studies were accidents, falls, assaults, and iatrogenic causes, and their incidence varies among different studies^9,19^. Generally, the current investigation has coincided with those whose results showed road traffic accidents (RTA) were representing the prominent factors causing injuries, followed by falls and assaults. In particular to angle fractures, they considerably caused by road traffic accidents and assaults, especially the left angle of the mandible, whereas condylar fractures were mostly resultant of falls. These results were in trends to given research literature^9,19^. Among patients in this study, angle fractures were observed to be isolated fractures rather than other fractures, while condylar fractures were usually in associations with other fractures. These variations were on par with prior studies^11^.

Another factor that increases the risk of the angular and condylar fractures is the existence or lack of occlusal support. A multi-center study investigated the prevalence of occlusal support in fractures of the lower jaw (angle and condyle); the sample included 298 patients with mandibular fractures. This study showed that the risk of condyle fractures was significant in patients lacking the occlusal support, while the risk of angle fractures was significant in patients with occlusal support. Our results revealed an equal significance concerning the presence or lack of occlusion^12^. The molars are the keystones of the occlusal support. The investigations carried out by previous studies have shown that the absence of occlusion will lead to mandibular fractures^13,20^. The current study reported that the deficiency of occlusal support would lead to fractures on the condyle. As the occlusion is less when the third molar is missing, this might result in poor occlusal support, leading to a more ability of movement when the force of impact is subjected toward the lower jaw, resulting in condylar fracture. Alternatively, when occlusion is present, and the third molar is in place, this might lead to fractures of the angle. Keeping the M3 in the proper position will enhance the occlusal support.

Studies showed constrained proof to express the effect of lower M3 on the angular and condylar fractures. Not many of them had yet explained the correlation of mandibular fractures to the different positions, inclinations, and root configuration of the lower M3^19^. The current study, in correspondence with part of the past investigations, confirms that the existence of lower M3 is significantly related to angular fracture, while the lack of lower M3 is significantly related to condylar fractures^21^. This result has been proved by a comprehensively meta-analysis published in 2017 which affirmed through a cumulative meta-analysis that OR varied only from 3.10 to 3.38, after the inclusion of eleven papers published between 2005 to 2017^7,8^. On the contrary, some studies have stated that the existence of the lower M3 has no relation in influencing angle or condyle fractures^22^. Authors of previous literature achieved various conclusions, and upon that, they recommended the need for further researches to ensure the results regarding the angulations and positions of M3. Based on Pell and Gregory's types of impactions, the current study found a higher risk of angular fractures accompanied with type B and type A positions. While for the condylar fractures, type C was in higher incidence than type A and B. This was equivalent to studies presented by Armond and Nogami^7,11^. According to Pell and Gregory's classes of impactions, the presenting study noticed a significant relationship between class I with an incidence of angular fractures followed by class II, while for condylar fractures, class II and class III were more associated. This was in relatively accordance with previous studies done by Iida, which found that these fractures were more present in class I, although there is less vulnerability to mandibular angle fractures compared to class III^23^. In contrast, other literature presented by Heulke, and Ma'aita said that deeply impacted M3 Class III position is more present with a high risk of mandibular angular fracture^6,24^. Moreover, according to Choi, which reported that lower M3 has the most favorable position of the third molar for angle fracture is class II^25^. Checking the Winter's classification of M3 angulations, this study observed that the mesioangular inclinations of lower M3 were correlated to the increased incidence of angular fractures. On the contrary, vertical inclinations have occupied the highest incidence of condylar fractures. These findings were in line with earlier studies^14^. Whereas, in conflict with other studies such as Meisami, who declared that mesioangular and distoangular inclinations are more associated with mandibular fractures^26^.

Finally, conclusions about third molar retaining or removing are still controversial. Although some studies suggest that the third molar can cause complications after mandibular angle fracture treatment^27,28^, prophylactic extraction of the lower M3 is a questionable matter. There is no reliable proof of whether to advocate or reject the prophylactic removal of the lower M3^11^. The option to remove or retain lower M3 to diminish the mandibular angular fractures' danger requires many considerations as the mandibles lacking M3s may lose the occlusal support and be more susceptible to condylar fractures^21^. Treatment of the condylar fractures usually faces more challenges in vision, surgical access, reduction, and fixation due to its small size and anatomical site. Post-operative complications may occur more in condylar fractures rather than angular ones.

There are some limitations in this study that must be considered in the final conclusions. The study design is cross-sectional. Thus, it is not possible to ensure the causality relation between occlusal support and the type of mandibular fracture. The other limitation is that this study was not a multicenter study, thus the population characteristics may not represent the universality of mandibular fractures.

In conclusion, taking account the limitations disclosed above, the lack of occlusal support seems to be more associated with condyle fractures than the presence of occlusal support and Class IB of M3 impactions are correlated with angle fractures while Class IIC are associated with condyle fractures.
